# Aortic dissection induced by licorice tablets overdose: a Case Report

**DOI:** 10.3389/fphar.2025.1610147

**Published:** 2025-08-29

**Authors:** Xin Luo, Yuhan Wang, Bin Liu, Duyi Zhang, Yanfen Chai, Lijun Wang

**Affiliations:** ^1^ Department of Emergency Medicine, Tianjin Medical University General Hospital, Tianjin, China; ^2^ College of Chemistry, Tianjin Normal University, Tianjin, China; ^3^ Department of Emergency Medicine, Tianjin Third Central Hospital, Tianjin, China

**Keywords:** liquorice, glycyrrhizic acid, aortic dissection, poisoning, pseudohyperaldosteronism

## Abstract

**Objective:**

To investigate the pathogenesis of aortic dissection induced by licorice tablet poisoning.

**Methods:**

A case of aortic dissection caused by poisoning by overtaking licorice tablets was reported. The patient’s medical history, laboratory examination, imaging results, and treatment process were analyzed, and the relevant literature was reviewed.

**Results:**

This case suggests that licorice poisoning is a potential risk factor for aortic dissection, which needs to be paid attention to in clinical practice. In the face of acute poisoning, in addition to paying attention to the conventional treatment measures of acute poisoning, we should pay more attention to the pharmacological mechanism of drugs and their induced complications to avoid subjective judgment and reduce misdiagnosis and abuse.

## 1 Introduction

Compound licorice tablets are commonly used antitussive and expectorant drugs. Each tablet contains 112.5 mg of licorice extract powder, 4 mg of opiate powder, 2 mg of camphor, 2 mg of anisole oil, and 2 mg of sodium benzoate. In addition to allergic reactions, nausea and diarrhea, the common adverse reactions of licorice tablets can also cause hypertension, water and sodium retention, hypokalemia and other water and salt metabolism disorders (Huo et al., 2021). The existing literature primarily focuses on the clinical management of hypokalemia and hypertension induced by long-term drug use (Albahlawan et al., 2024), with limited reports addressing acute toxic reactions resulting from drug overdose. The authors recently encountered a case of aortic dissection following an overdose of licorice tablets. Based on this case and a review of relevant literature, this article aims to provide insights for the diagnosis and treatment of such poisonings, as well as the early recognition of associated cardiovascular complications.

## 2 Clinical data

**FIGURE 1:**
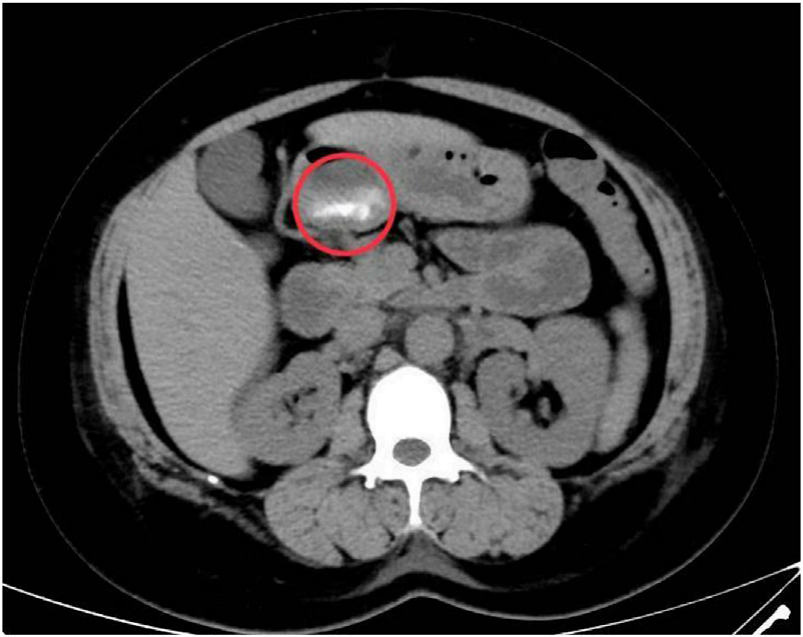
Abdominal CT.

**FIGURE 2:**
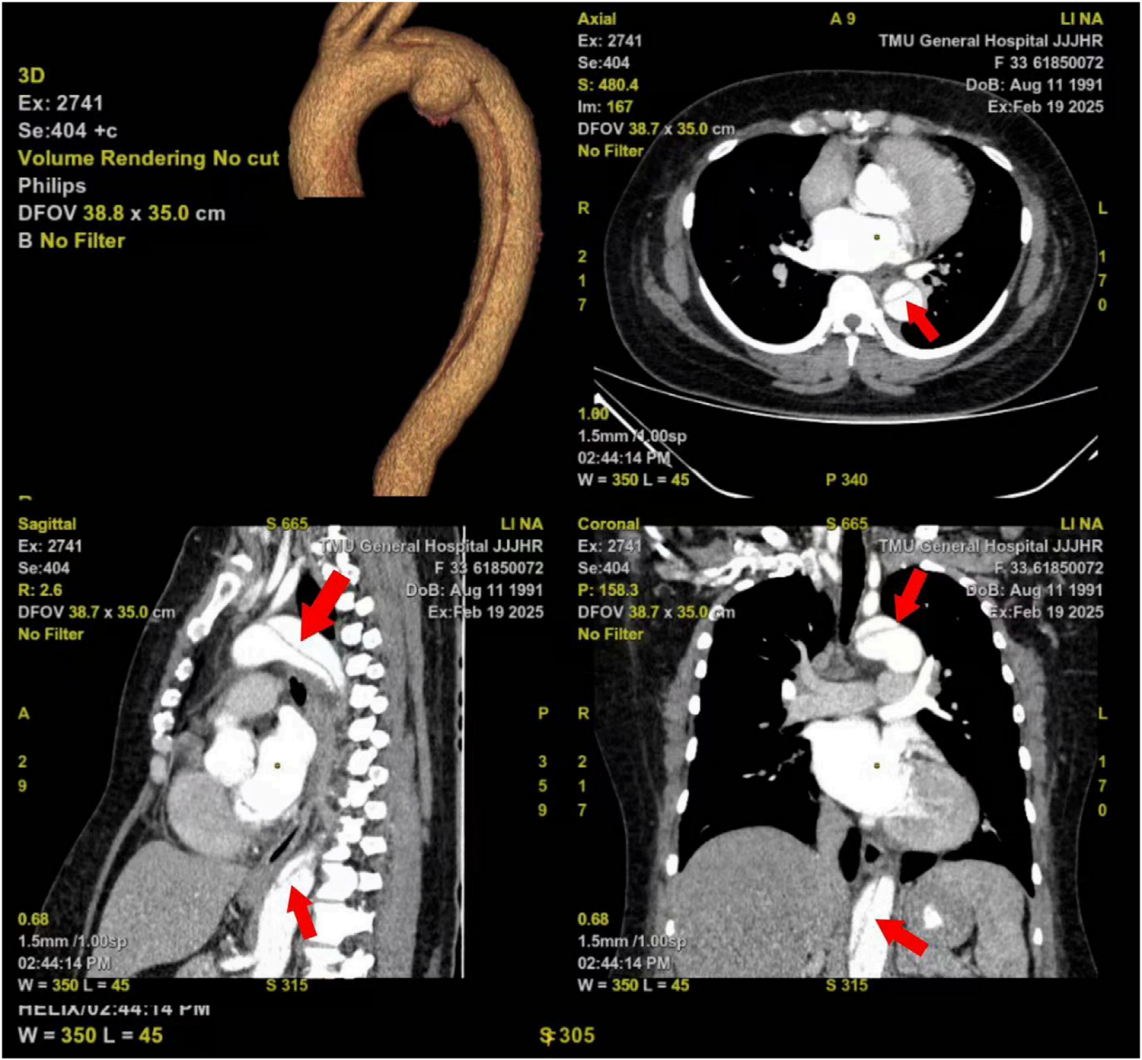
CTA of the aorta.

A 34 years old female was admitted to local hospital at 21:00 on 18 February 2025, due to “abdominal pain with vomiting for 4 h after taking 300 licorice tablets”. She had a history of hypertension for more than 10 years, with a maximum blood pressure of 200/100 mmHg, and poor blood pressure control. The patient had a 3-year history of coronary heart disease and chronic licorice tablet dependence. The patient has a smoking history and no drinking history. The average cigarette consumption is approximately 25 cigarettes per day. On admission, she was conscious but exhibited facial grimacing, blood pressure of 202/131 mmHg, heart rate 103 bpm, and SpO_2_ 96%. Hematological examination is shown in Table 1, and abdominal CT imaging revealed no significant abnormalities. Following symptomatic and supportive treatments, including gastric lavage and fluid infusion, the patient continued to experience abdominal pain. She was subsequently referred to our emergency department at 4:00 on the next day. The pain was described as a persistent tearing sensation that progressively intensified, accompanied by radiating pain to the shoulder, nausea, and vomiting. Upon admission, her vital signs were as follows: temperature 37.2 °C, heart rate 96 bpm, and blood pressure 232/143 mmHg. The patient was alert but irritable, with coherent responses. Physical examination revealed a soft abdomen and general abdominal tenderness, no obvious rebound pain and muscle tension, and percussion tenderness over kidney region (−). Hematological examination is shown in Table 1. Follow-up abdominal CT demonstrated retained tablets in the gastric lumen (Figure 1), with no other significant abnormalities identified. Considering persistent severe abdominal pain and progressively rising D-dimer levels (Table 1), computed tomographic angiography (CTA) of the aorta revealed a Stanford classification type B aortic dissection with an identifiable tear in the aortic arch region. The dissection extends to the abdominal aorta and right common iliac artery, involving the origin of the celiac trunk. The superior mesenteric artery and right renal artery arise from the true lumen, while the left renal artery, inferior mesenteric artery, and left common iliac artery originate from the false lumen (Figure 2). The patient underwent emergency Endovascular exclusion with single-branch stent graft placement at another hospital, and achieved satisfactory postoperative recovery.

**TABLE 1:** Laboratory results of patients at different times.

	02–18 21:00	02–19 04:00	02–19 17:00
Physical examination			
BP(mmHg)	202/131	232/143	
HR (bpm)	103	96	
SpO_2_(%)	96	97	
Blood routine			
WBC(*10^9^/L)	25.03	19.41	14.94
N%	87.1	86.8	83.2
L%	8.0	8.5	11.6
RBC(*10^12^/L)	3.93	3.77	3.39
Hb(g/L)	123	120	108
PLT (*10^9^/L)	210	125	107
Electrolytes			
K^+^ (mmol/L)	2.88	3.3	2.9
Na^+^(mmol/L)	133.4	131	133
Cl^−^(mmol/L)	96.5	92	98
Coagulation function			
PT (sec)	—	1.27	13.1
APTT (sec)	—	40.8	32.8
FIB(g/L)	—	—	4.35
D-Dimer (mg/L)	1.977	4.460	>10.00
Arterial blood gas analysis			
PH		7.467	
PaO_2_(mmHg)		79	
PaCO_2_(mmHg)		38.1	

## 3 Discussion

Licorice tablets can elevate blood pressure through multiple mechanisms: (1) Glycyrrhizic acid and its metabolites, such as glycyrrhetinic acid, inhibit the enzyme 11β-hydroxysteroid dehydrogenase type 2 (11β-HSD2), which prevents the conversion of cortisol into inactive metabolites. This leads to cortisol accumulation and subsequent activation of mineralocorticoid receptors in renal collecting duct cells, mimicking the effects of aldosterone and causing pseudohyperaldosteronism. In the kidney, this results in abnormal activation of ROMK, ENaC, and AQP2 channels, exacerbating water and sodium retention as well as potassium loss, ultimately leading to hypertension with hypokalemia. During the initial visit, the patient’s serum potassium level was measured at 2.88 mmol/L, aligning with this pathophysiological mechanism. (2) Additionally, glycyrrhiza metabolites enhance vasoconstrictor responses and inhibit endothelial nitric oxide synthesis by activating the endothelin system, directly increasing arterial tension and worsening hypertension (Ceccuzzi et al., 2023).

Acute aortic dissection (AD) represents a critical cardiovascular emergency with substantial mortality risk, demonstrating an initial mortality rate approaching 40%. This life-threatening condition is pathologically characterized by blood extravasation through an intimal tear into the medial layer of the aortic wall, creating a false lumen through longitudinal separation of the vascular wall layers. The development of AD is influenced by multiple factors, with hypertension being the most prevalent risk factor, present in approximately 75%–80% of AD patients. Hypertension primarily contributes to AD pathogenesis by promoting atherosclerotic changes and stimulating the production of matrix metalloproteinases (MMPs) and pro-inflammatory cytokines, which collectively impair the vascular wall and reduce its elasticity (Sayed et al., 2021; Yuan et al., 2022). Therefore, the main trigger of the aortic dissection after this patient took large doses of licorice tablets may be due to the decrease in vascular elasticity caused by long-term hypertension and poor blood pressure control, combined with the sharp increase in blood pressure, which eventually led to the tear of the aortic intima, thus causing the aortic dissection. Moreover, aortic dissection can result in severe pain. This pain triggers the body’s stress response, activates the hypothalamic-pituitary-adrenal axis and the sympathetic nervous system, and reflexively causes a rise in blood pressure. When the pain persists over an extended period, the response of pressure receptors to the blood pressure increase induced by the pain often diminishes, ultimately leading to a sustained elevation in blood pressure (Rivasi et al., 2022). This vicious cycle could be an additional potential factor contributing to the rapid progression of aortic dissection in this patient.

The reasons for the delay in diagnosis of this patient can be attributed to the following factors: (1) Aortic dissection often presents with non-specific clinical manifestations, which can easily lead to confusion with more common diseases and consequently result in misdiagnosis. According to existing studies, over 30% of cases experience either missed or delayed diagnosis during the initial clinical evaluation (Yang et al., 2023). Due to the long-term addictive nature of licorice tablets, withdrawal from the drug may lead to symptoms such as agitation (Huo et al., 2021). The patient had visited the emergency department multiple times due to abdominal pain after overusing licorice tablets. During this visit, the patient exhibited abdominal pain, irritability, and incoherent speech, resembling a condition akin to “hysteria,” which complicated the doctor’s accurate assessment of the situation. (2) Before diagnosis, the patient’s abdominal CT and ultrasound revealed no abnormalities. However, transthoracic echocardiography has limited sensitivity for aortic dissection, particularly in type B dissections. Compared to CTA, conventional CT has a higher rate of missed diagnoses and false negatives. Additionally, while D-dimer testing exhibits high sensitivity, its specificity is relatively low for aortic dissection (Chen et al., 2022; Kesieme et al., 2024). Therefore, CT and ultrasound examination do not find obvious abnormalities, but the patient has persistent severe abdominal pain and is accompanied by hypertension. The plasma D-dimer level was significantly increased in the case of persistent severe abdominal pain and hypertension. The possibility of thrombotic disease should be considered. An elevated D-dimer level within 24 h after symptom onset has a sensitivity of 94%, especially at a cutoff above 0.5 μg/mL, which should be used to make an impact diagnosis (Sayed et al., 2021). Therefore, in the face of chronic hypertensive patients with acute chest pain, abdominal pain and elevated D-dimer, the possibility of acute aortic dissection should be given priority.

In cases of acute poisoning, emergency physicians primarily consider interventions such as gastric lavage, catharsis, antidote administration, and blood purification, often overlooking potential complications. This case highlights the necessity of not only focusing on conventional treatment protocols for acute poisoning but also emphasizing the pharmacological mechanisms of therapeutic agents and their associated complications. Such an approach can minimize subjective clinical judgments and reduce the incidence of misdiagnosis and inappropriate treatment.

## Data Availability

The original contributions presented in the study are included in the article/Supplementary Material, further inquiries can be directed to the corresponding author.
